# Manipulation of Luminescence via Surface Site Occupation in Ln^3+^-Doped Nanocrystals

**DOI:** 10.1021/jacs.4c00052

**Published:** 2024-04-16

**Authors:** Rui Shi, Litian Lin, Zijun Wang, Qilin Zou, Anja-Verena Mudring

**Affiliations:** † Intelligent Advanced Materials, Department of Biological and Chemical Engineering and iNANO, Aarhus University, Aarhus C 8000, Denmark; ‡ State Key Laboratory of Rare Metals Separation and Comprehensive Utilization, Guangdong Provincial Key Laboratory of Rare Earth Development and Application, Institute of Resources Utilization and Rare Earth Development, Guangdong Academy of Sciences, Guangzhou 510651, China; § IMRB, Université Paris Est Créteil, INSERM U955, CNRS, EMR 7000, 94010 Créteil, France; ∥ Laboratoire de Physique de la Matière Condensée, Ecole Polytechnique, CNRS, IP Paris, 91128 Palaiseau, France; ⊥ Department of Physics, Umeå University, Linnaeus väg 24, 901 87 Umeå, Sweden

## Abstract

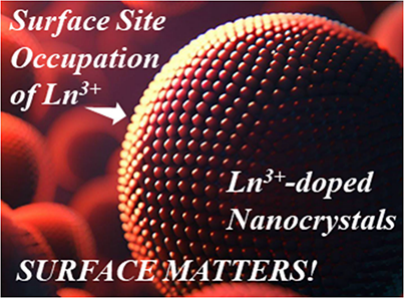

Ln^3+^-doped (Ln = lanthanide) nanocrystals are garnering strong interest for their potential as optical materials in various applications. For that reason, a thorough understanding of photophysical processes and ways to tune them in these materials is of great importance. This study, using Eu^3+^-doped Sr_2_YF_7_ as a well-suited model system, underscores the (not unexpected) significance of surface site occupation of Ln^3+^ and also challenges the prevailing views about their contribution to the luminescence of the system. High-temperature cation exchange and epitaxial shell growth allow nanocrystals to exclusively feature Eu^3+^ residing at the surface or in the interior, thereby separating their spectral responses. Meticulous experiments reveal that nanocrystals with high doping concentrations exhibit luminescence primarily from surface Eu^3+^, in contrast to the popular belief that luminescence from surface Ln^3+^ is largely negligible. The present study shows, on the one hand, the necessity to revise common ideas and also reveals the potential for manipulating the luminescence of such materials through an, until now, unperceived way of surface engineering.

## Introduction

Technological advancements create an increasing demand for high-performance optical nanomaterials.^1^ Ln^3+^-doped (Ln = lanthanide) optical materials are ideal candidates as the intraconfigurational transitions within the well-shielded 4f orbitals of Ln^3+^ endow them with luminescence properties unattainable from other materials,^2,3^ as shown by plenty of examples.^4,5^

Yet, there persist many challenges that hamper the application potential of Ln^3+^-doped luminescent nanocrystals (NCs). Despite intense research efforts, there is still not a complete understanding of their luminescent behaviors.^6,7^ Although generally the interpretation of spectroscopic features of Ln^3+^-doped (bulk) materials is very well established,^8^ the accumulation of new observations associated with the surface of NCs calls for improving, if not revising, the current view.^9−13^ The drastic rise in the surface-to-volume ratio of NCs over their bulk counterparts increases the probability that dopant Ln^3+^ resides at the particle surface (termed surface Ln^3+^). The puzzle is that there is a diversity of diverging opinions regarding the contribution of surface Ln^3+^ to the overall luminescence of the NCs. Some hold the view that both Ln^3+^ at the NC surface and in its interior (termed bulk Ln^3+^) contribute to the overall luminescence, but as they have different environments, they need to be considered separately.^14,15^ Others claim that attention to surface Ln^3+^ is not needed as luminescence from those ions is largely quenched.^16,17^ The views go even further, suggesting that it is reasoned that surface Ln^3+^ will experience such severe luminescence quenching that it constitutes an optically silent layer outward of the NC.^18^ This opinion is commonly adopted in the case of Ln^3+^-doped upconverting NC as the de-excitation of upconverted Ln^3+^ such as Er^3+^ is strongly mediated by surface-related vibronic quenching.^19^

The divergence between different views on the contribution of surface Ln^3+^ to the luminescence properties of these materials perplexes and, consequently, a more thorough understanding needs to be evidently established, which motivated this study.

**Figure 1:**
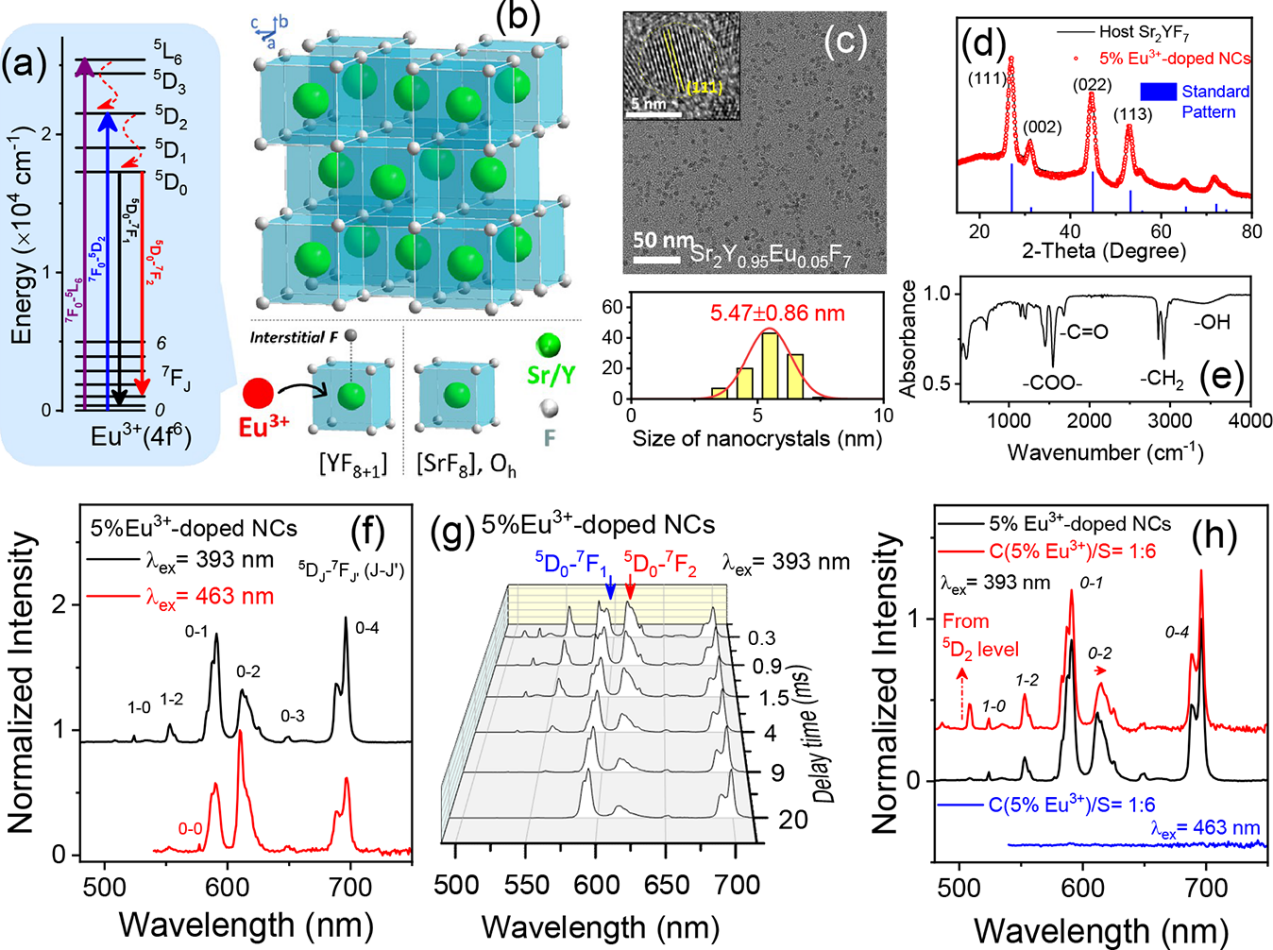
(a) Energy level diagram of Eu^3+^ with the 4*f*^6^ configuration. (b) Illustration of the Sr_2_YF_7_ crystal structure. Compared to the cubic [SrF_8_] coordination polyhedron with an ideal O_h_ symmetry, the residence of Y^3+^ and Eu^3+^ at the same Wyckoff 4a site results in a F^–^ ion located at the nearest-neighbor interstitial space. (c) TEM image of surfactant-capped Sr_2_Y_0.95_Eu_0.05_F_7_ NCs of approximately 5 nm diameter. Inset: high-resolution TEM image of a single NC. (d) PXRD patterns of undoped and Eu^3+^-doped Sr_2_YF_7_ NCs. (e) Representative IR spectrum of surfactant-capped NCs. (f) Normalized emission spectra of 5%Eu^3+^-doped Sr_2_YF_7_ NCs upon different excitations, with different spectral features collected for the Eu^3+^ emission. (g) Time-resolved emission spectra of 5%Eu^3+^-doped NCs upon 393 nm excitation with delay times from 0.3 to 20 ms. The ^5^D_0_-^7^F_1_ and ^5^D_0_-^7^F_2_ transitions of Eu^3+^ show divergent decay trends with the extension of the signal acquisition delay. (h) Comparison of the normalized emission spectra of the Eu^3+^-doped core and core–shell Sr_2_YF_7_ NCs (with a nominal molar ratio of luminescent-core and inert-shell compositions equal to 1:6) upon different excitations.

To properly delineate the contributions of surface and bulk Ln^3+^ in the NCs, a suitable study system and an appropriate experimental strategy must be established. The absence of translational periodicity makes conventional X-ray diffraction approaches challenging.^20^ The luminescence of Eu^3+^ offers an opportunity for tackling this puzzle^21^ as the electronic transitions between different 4*f* energy levels of Eu^3+^ (Figure 1a) are strongly influenced by the local coordination,^22^ which allows for the distinction between occupation at surface and bulk sites. However, for this approach to be successful, the local environments of surface and bulk Eu^3+^ in the NCs have to be significantly different as previous attempts vividly illustrate.^10,14,23^ Thus, a proper host structure ought to be chosen to ensure that Eu^3+^ at the surface or in the interior possesses recognizably distinct spectroscopic features that can be clearly distinguished from each other.^24^ In addition, the preparation of such Ln^3+^-doped NCs for such investigations needs to be highly reproducible and robust^10,25−27^ for compelling conclusions to be drawn.^28^

In this context, Ln^3+^-doped Sr_2_YF_7_ ternary fluorides come into focus.^29^ Recent work has shown their luminescence efficiency being better than that of popular hexagonal-phase NaLnF_4_ when the size of the NCs is less than 10 nm.^30^ Sr_2_YF_7_ crystallizes in a superstructure related to fluorite (CaF_2_), where Sr^2+^ and Y^3+^ occupy the Ca^2+^ cationic site of the parent CaF_2_ structure, which is coordinated by eight F^–^ ions in the form of a cube (Figure 1b). While the isovalent substitution of Ca^2+^ with Sr^2+^ does not lead to changes from a structure chemical perspective, does the aliovalent substitution of Ca^2+^ with the trivalent Y^3+^ call for extra F^–^ for charge compensation. This F^–^ preferentially locates at the nearest-neighbor interstitial space,^31^ capping one of the square faces of the [YF_8_] cube, thereby distorting the local symmetry from the ideal O_h_ symmetry. In the cubic structure, there is only one crystallographically independent cation site (Wyckoff 4a position) available for substitution when the dopant Ln^3+^ (i.e., Eu^3+^ in the current case) is introduced, and Sr and Y occupy this site with different ratios. The Y^3+^ surface sites, on the other hand, distort strongly from the O_h_ symmetry due to the incomplete coordination environment. Thus, the substitution of Eu^3+^ at these two Y^3+^ sites can be distinguished based on the difference in luminescence 4f-4f transitions of Eu^3+^ due to the stronger relaxation of the selection rule with lowered site symmetry.^32^ For those reasons, Eu^3+^-doped Sr_2_YF_7_ is chosen in this work to unveil the impact of surface Ln^3+^ on the luminescence properties of the NCs.

## Results and Discussion

Thermal decomposition of appropriate amounts of strontium and lanthanide trifluoroacetate in ODE (1-octadecene) in the presence of OA (oleic acid) and OM (oleylamine) (see Methods for the experimental details given in the Supporting Information) yielded ∼5 nm-sized Eu^3+^-doped Sr_2_YF_7_ NCs with a uniform spheroidal morphology, as confirmed by transmission electron microscopy (TEM) (Figure 1c). HRTEM (high-resolution TEM) of selected NCs shows lattice fringes that can be associated with the {111} set of planes of an undistorted CaF_2_-type-related cubic structure for Sr_2_YF_7_ (Figure 1c). This is further confirmed by powder X-ray diffraction (PXRD) analysis (Figure 1d). No indication of the formation of ordered superstructures or structural distortions can be detected. The occurrence of high-frequency vibrational modes of organic groups in the IR spectrum of the sample (Figure 1e) demonstrates the surface binding of surfactants OA and OM.

Exciting 5%Eu^3+^-doped Sr_2_YF_7_ NCs with 393 nm corresponding to the ^7^F_0_-^5^L_6_ transition of Eu^3+^ (Figure 1a) reveal in the emission spectrum the Eu^3+^^5^D_0_-^7^F_1_ magnetic-dipole transition (Δ*J* = 1) being dominant over the forced ^5^D_0_-^7^F_2_ electric-dipole transition (Δ*J* = 2), together with an intense Eu^3+^^5^D_0_-^7^F_4_ transition observed (Figure 1f). The former reveals a minor relaxation of the selection rule as the Δ*J* = 2 transition should have been strictly forbidden with Eu^3+^ residing at a site with O_h_ symmetry,^33^ and the latter indicates a special distortion of the local Eu^3+^ coordination.^22,34^ A similar spectral feature has been recorded for Eu^3+^-doped Ca_3_Sc_2_Si_3_O_12_,^35^ in which Eu^3+^ resides at a distorted cubic site with actual D_2_ symmetry. These findings agree with the expected substitution of Eu^3+^ at the Y^3+^ site in Sr_2_YF_7_. Moreover, weaker emissions originating from the Eu^3+^^5^D_1_ level were also recorded. When the excitation wavelength was changed to 463 nm, corresponding to the ^7^F_0_-^5^D_2_ transition of Eu^3+^ (Figure 1a), different spectral features were detected. Here, the Eu^3+^^5^D_0_-^7^F_2_ emission dominates, indicating emission from a Eu^3+^ site that strongly deviates from inversion symmetry (unlike that expected for the high-symmetric Y^3+^ site in the interior) (Figure 1f). The anomaly brought about by this extra site occupation is visible not only in the steady-state spectra (Figure S1a,b) but also in the luminescence decay dynamics (Figure S1c). Upon 393 nm excitation, the relative intensities of different Eu^3+^ 4f-4f emissions vary differently with the extension of the signal acquisition delay (Figure 1g). Aside from the rapid decays from the Eu^3+^^5^D_1, 2_ levels as generally observed, the collection of divergent trends in the ^5^D_0_-^7^F_1_ and ^5^D_0_-^7^F_2_ transitions is puzzling, given that the intensity branching ratio of emission from the same 4f excited level of an emitting Eu^3+^ species should have stayed constant, as its ratio is determined by the coordination environment of Eu^3+^.^36^ Intriguingly, these anomalies can be eliminated when a luminescent-inert Sr_2_YF_7_ shell is epitaxially grown on the 5% Eu^3+^-doped NCs (Figures 1h and S1d). In this case, the spectral shape of the Eu^3+^^5^D_0_ emission is largely preserved upon 393 nm excitation, with a clear enhancement of emissions from the ^5^D_1, 2_ levels. No visible Eu^3+^ luminescence can be detected upon 463 nm excitation of the core–shell NCs. These observations reveal that the luminescence of Eu^3+^-doped Sr_2_YF_7_ NCs is significantly affected by the presence of surface Eu^3+^.

**Figure 2:**
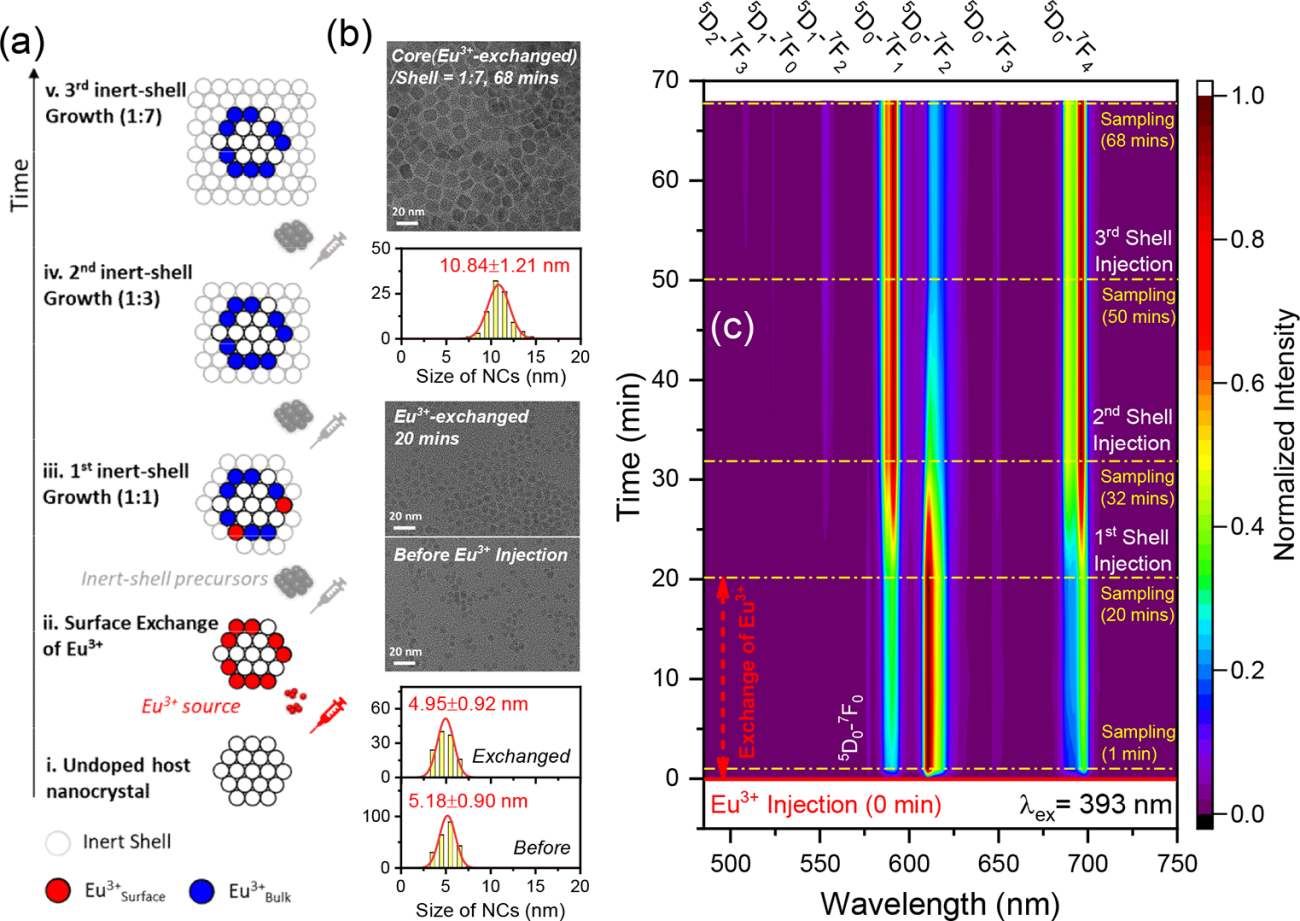
(a) Schematic diagram of the experimental flow for Eu^3+^ surface exchange and the subsequent conversion of surface Eu^3+^ to bulk Eu^3+^ by inert-shell growth. (i) Undoped host NCs; (ii) Eu(III) oleate dispersed in 1-octadecene was hot-injected into the reaction mixture in one shot, triggering the Y^3+^-to-Eu^3+^ cation exchange; (iii–v) Precursors to generate a Sr_2_YF_7_ inert-shell were hot-injected shot-by-shot into the reaction mixture ensuring the epitaxial shell growth on the Eu^3+^-exchanged NCs. Aliquots were extracted from the reaction mixture at the end of each stage. (b) TEM images and the size distributions of representative samples before Eu^3+^ injection, Y^3+^-to-Eu^3+^ cation-exchanged NCs, and NCs with sufficiently thick inert-shell grown. (c) Color contour of the normalized emission spectra of Eu^3+^ in NCs extracted from the reaction mixture at different stages upon 393 nm excitation. The moment of injection of the Eu^3+^ source is defined as the time reference of 0 min.

Understanding the impact of surface site occupation on the luminescence of Eu^3+^-doped Sr_2_YF_7_ NCs requires a thorough knowledge of the spectroscopic features of Eu^3+^ substituting at various sites in the system. Unfortunately, this information is challenging to derive from the direct-doped samples because of the confounding caused by the crosstalk of the spectral responses of crystallographically different Eu^3+^ and their interactions. Thus, a special synthetic approach was developed to circumvent this difficulty (Figure 2a). Eu^3+^ in high concentration is initially introduced at the surface of the undoped NCs (Figure 2a,i) through high-temperature cation exchange (Figure 2a,ii) and subsequently converted to residing inside the NC by epitaxial inert-shell growth (Figure 2a,iii–v). In this way, NCs exclusively featuring surface (Figure 2a,ii) or bulk Eu^3+^ (Figure 2a,v) are obtained. No significant change in size or morphology of NCs is observed to occur in the exchange process (Figure 2a,ii,b). In comparison, a spheroidal-to-cuboidal change in NC morphology is observed as the shell growth proceeds (Figure 2a,iii–v,b, top), with the size of NCs finally increasing to around 11 nm. The spectral features of samples collected at the end of each stage were recorded (Figures S2 and S3). In contrast to the expected nonluminescence of the undoped sample, intense luminescence that has to originate from surface Eu^3+^ is observed in the sample that is treated for 1 min of the Y^3+^-to-Eu^3+^ cation exchange procedure (Figure 2c). This luminescence is dominated by the ^5^D_0_-^7^F_2_ emission, and the spectrum is distinctly different from the luminescence spectrum of Eu(III) oleate used for the exchange (Figure S2c,d). Little impact on the spectral features can be seen by extending the exchange duration from 1 to 20 min. Subsequent growth of an inert-shell alters the spectral features (Figure 2c): the initially dominant ^5^D_0_-^7^F_2_ line of Eu^3+^ becomes weaker and the ^5^D_0_-^7^F_1_ line gains in relative intensity until it becomes dominant, as observed in the core–shell NCs where all Eu^3+^ resides in the core and in agreement with a higher symmetric site.

**Figure 3:**
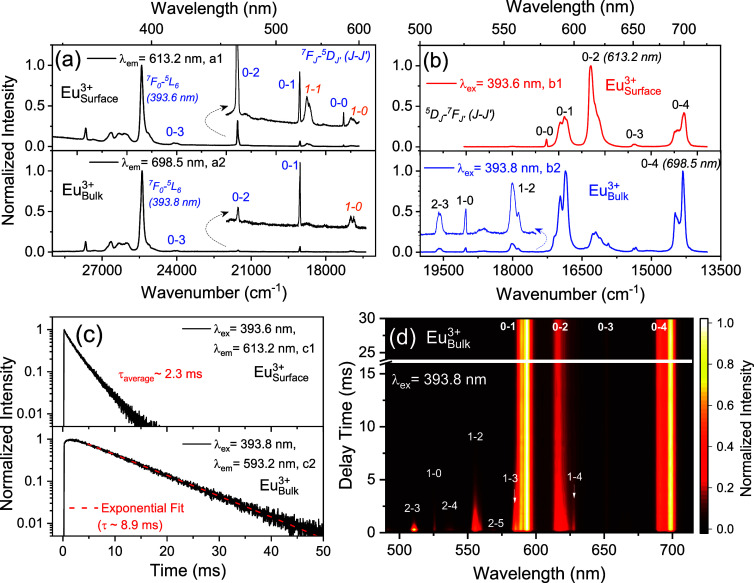
(a) High-resolution excitation spectra of Eu^3+^ surface-exchanged (20 min) and sufficiently thick inert-shell grown NCs (68 min) obtained by monitoring the Eu^3+^^5^D_0_ emissions. The enlargements of spectra in the range of 16,000–22,000 cm^–1^ are given as insets. (b) Corresponding emission spectra of surface and bulk Eu^3+^ in the representative samples upon excitation of the Eu^3+^^7^F_0_-^5^L_6_ transition. The terms and the corresponding wavelengths of relevant 4f-4f electronic transitions of Eu^3+^ are indicated. (c) Decay dynamics of the ^5^D_0_ luminescence of surface and bulk Eu^3+^ in the representative NCs. Compared to the nonexponential luminescence decay from the ^5^D_0_ level of surface Eu^3+^, a longer luminescence decay from the ^5^D_0_ level of bulk Eu^3+^ is observed together with the initial rise in the decay curve. (d) Color contour of the normalized TRES (time-resolved emission spectrum) of bulk Eu^3+^ upon excitation of the Eu^3+^^7^F_0_-^5^L_6_ transition (393.8 nm).

Valuable information can be extracted from the spectral fine structures of different Eu^3+^ species. Although similar responses are found in the range above 450 nm of the excitation spectra for surface and bulk Eu^3+^ by monitoring their ^5^D_0_ emissions (Figure 3a), their spectral features in the long-wavelength range (450–625 nm) are different (Figure 3a, insert). In contrast to the mere observation of the ^7^F_0_-^5^D_1_ line, with the ^7^F_0_-^5^D_2_ and ^7^F_1_-^5^D_0_ lines scarcely discernible in the spectrum of bulk Eu^3+^, an intense ^7^F_0_-^5^D_2_ excitation of surface Eu^3+^ far more than its ^7^F_0_-^5^D_1_ line is detected, along with a sharp ^7^F_0_-^5^D_0_ signal. The signals of the ^7^F_1_-^5^D_1_ and ^7^F_1_-^5^D_0_ lines are also visible in the spectrum of surface Eu^3+^-containing NCs, with the former hypersensitive one observed due to the relaxation of the selection rule.^22^ Distinct differences were also recorded in the emission spectra for the Eu^3+^ surface and bulk containing NCs (Figure 3b). The emission spectrum recorded for surface Eu^3+^-containing NCs exhibits clear dominance of the ^5^D_0_-^7^F_2_ emission over the ^5^D_0_-^7^F_1_, and one ^5^D_0_-^7^F_0_ line is seen at around 579 nm. Comparison of the emission spectra recorded for surface Eu^3+^-containing NCs by fine-tuning the excitation wavelength allows us to exclude the presence of different surface Eu^3+^ sites with different local environments (Figure S4). A detailed inspection reveals a 3-fold splitting of the ^5^D_0_-^7^F_1_ transition, pointing to the total removal of crystal field degeneracy (Figure S5a). Together with the appearance of the ^5^D_0_-^7^F_0_ transition, it is demonstrated that the site symmetry of surface Eu^3+^ is heavily distorted, such as toward C_1_ or *C*_*2*_ symmetry.^37^ Moreover, the absence of luminescence from the ^5^D_1, 2_ levels is in agreement with Eu^3+^ being exposed to an environment of organic surfactants, which leads to a severe nonradiative relaxation of these levels.^9^

In comparison, the emission spectrum of solely bulk Eu^3+^-containing NCs shows dominant ^5^D_0_-^7^F_1, 4_ emissions over the ^5^D_0_-^7^F_2_ one, along with the emissions from the ^5^D_1, 2_ levels. These emissions are regulated by the selection rule, with the Δ*J* = 1 lines being more intense. Some of them overlap with the luminescence from the ^5^D_0_ level. The removal of overlap by delaying the signal acquisition (*t*_delay_ = 10 ms) unveils both the 3-fold splitting of the ^5^D_0_-^7^F_1_ and ^5^D_0_-^7^F_2_ transitions of bulk Eu^3+^ (Figure S5b). Together with the absence of ^5^D_0_-^7^F_0_ transition in the spectra, it can be deduced that Eu^3+^ occupies the bulk Y^3+^ site with D_2_ symmetry (a similar site symmetry as in the Eu^3+^-doped garnet system mentioned above^35^). Also, the presence of different bulk Eu^3+^ sites with significantly different local environments can be excluded by comparing the emission spectra of bulk Eu^3+^-containing NCs recorded by fine-tuning the excitation wavelength (Figure S6).

The luminescence decay dynamics of surface and bulk Eu^3+^-containing NCs exhibit a stark difference (Figure 3c). A longer luminescence decay of 8.9 ms from the ^5^D_0_ level of bulk Eu^3+^ is seen as opposed to the nonexponential luminescence decay from the ^5^D_0_ level of surface Eu^3+^ that has an average lifetime of 2.3 ms. It mostly accords with the radiative probability of bulk Eu^3+^ being more constrained by the selection rule compared to that of surface Eu^3+^, with the latter likely being vulnerable to surface-related quenching effects. An initial rise in the decay curve of bulk Eu^3+^ is also seen, corresponding to a sluggish population from the upper 4f levels to the bulk Eu^3+^^5^D_0_ level. More details for understanding these features are available from the time-resolved emission spectra (TRES, Figures 3d and S7). The consecutive decays of the ^5^D_2_, ^5^D_1_, and ^5^D_0_ luminescence of bulk Eu^3+^ show that the selection rule constrains not only the radiative transition but also the nonradiative relaxation,^38^ resulting in a sluggish and sequential de-excitation between the adjacent levels (Δ*J* = 1) of bulk Eu^3+^.

These observations reveal that surface Ln^3+^ exhibits intriguing spectral features and luminescence dynamics that are unattainable from those of bulk Ln^3+^ in the same host crystal system, all of which are associated with the relaxation of the selection rule induced by the local coordination distortion on the surface of NCs. More intriguingly, surface Eu^3+^ in the current case even shows superior luminescence brightness compared to that of bulk Eu^3+^ (Figure S8), which will surely spark intensive research enthusiasm for regulating the luminescence properties of Ln^3+^-containing nanocrystals through a novel way of surface engineering. In addition, both the surface exchange and epitaxial shell growth experiments were conducted with a low exchange concentration of Eu^3+^ (Figure S9), as well as in different systems (Sr_2_GdF_7_, Sr_2_LuF_7_, and SrF_2_; Figure S10). We confirm the effectiveness of this investigation strategy in identifying the site occupation of Ln^3+^ in the nanocrystals by observing similar outcomes in all cases. Evidently, the mutual interference between the spectral responses of crystallographically different Ln^3+^ results in the unique luminescent behavior of Ln^3+^-doped NCs, as seen in 5% Eu^3+^-doped Sr_2_YF_7_ (Figure 1f,g).

**Figure 4:**
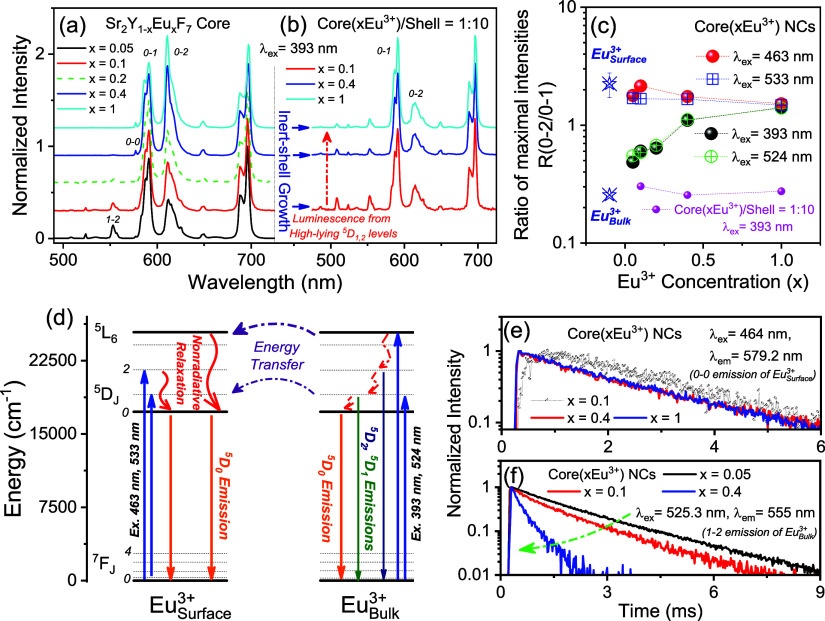
(a) Normalized emission spectra of Sr_2_Y_1–*x*_Eu_*x*_F_7_ (*x* = 0.05–1) NCs upon 393 nm excitation and (b) their counterparts with a successively grown inert-shell (with a nominal molar ratio of core and shell compositions equal to 1:10). The epitaxial inert-shell growth converts the spectral shape of the Eu^3+^^5^D_0_ emission to that of bulk Eu^3+^ completely in the Eu^3+^-doped nanocrystals with different doping concentrations, as well as the emergence of luminescence from the high-lying ^5^D_1,2_ levels of bulk Eu^3+^. (c) Dependence of the intensity ratio of the Eu^3+^^5^D_0_-^7^F_2_ and ^5^D_0_-^7^F_1_ emission maxima, the R value, on the Eu^3+^ concentration upon different excitations. The corresponding R values of surface and bulk Eu^3+^ as well as the Eu^3+^ in the samples with an inert-shell grown are also provided for comparison. (d) Schematic energy level diagrams of different Eu^3+^ in the system. The construction of the diagram relies on the precise determination of different 4f-4f transition energies of Eu^3+^ from the high-resolution spectra of the samples. The relevant processes of excitations, emissions of surface and bulk Eu^3+^, and energy transfer between surface and bulk Eu^3+^ are marked by arrows. (e) Decay dynamics of the ^5^D_0_-^7^F_0_ emission of surface Eu^3+^ and (f) that of the ^5^D_1_-^7^F_2_ emission of bulk Eu^3+^ in the NCs with different Eu^3+^ concentrations. In contrast to the nearly invariance of the ^5^D_0_ luminescence decay behavior of surface Eu^3+^, a noticeable acceleration of the luminescence decay from the ^5^D_1_ level of bulk Eu^3+^ upon excitation of the Eu^3+^^7^F_0_-^5^D_1_ transition is observed as the increase in Eu^3+^ concentration in the Eu^3+^-doped Sr_2_YF_7_ nanocrystals, which is associated with the energy transfer from bulk Eu^3+^ to surface Eu^3+^ in the system.

The impact caused by the surface site occupation of Ln^3+^ becomes more substantial in systems with high doping concentrations. Upon 393 nm excitation, a considerable enhancement of the Eu^3+^^5^D_0_-^7^F_2_ emission over its ^5^D_0_-^7^F_1_ one was detected in the emission spectra of Sr_2_Y_1–*x*_Eu_*x*_F_7_ NCs as the Eu^3+^ concentration increased from 5 to 100% (Figure 4a), while there was no notable change in the morphologic features and crystalline phase of NCs (Figure S11). The complete conversion of the spectral shape of the Eu^3+^^5^D_0_ emission to that of bulk Eu^3+^ in the samples with different Eu^3+^ concentrations after inert-shell coating excludes the contribution of Eu^3+^–Eu^3+^ cluster formation^31^ (Figure 4b). Intriguingly, this concentration-dependent variation in spectral features is excitation-wavelength-dependent (Figures 4c and S12), showing that this striking enhancement of the ^5^D_0_-^7^F_2_ emission is only observed when bulk Eu^3+^ is effectively excited (upon 393 and 524 nm excitations) in the system, in contrast to the consistent dominance of the ^5^D_0_-^7^F_2_ emission with the favored excitation of surface Eu^3+^ (upon 463 and 533 nm excitations), regardless of the Eu^3+^ concentration.

Looking at the energy level diagrams for surface and bulk Eu^3+^ (Figures 4d and S13) helps us to understand this impact of surface Eu^3+^ on the excited-state dynamics of the entire system. The excitation of surface Eu^3+^ leads to a predominant population of its ^5^D_0_ level since the high-lying ^5^D_1,2_ levels are rapidly de-excited through vibronic quenching from the surfactants.^7^ Radiative emission from the ^5^D_0_ level of surface Eu^3+^ is preferred over transferring energy to bulk Eu^3+^, even though the ^5^D_0_ levels of surface and bulk Eu^3+^ are largely resonant, as the ^7^F_0_-^5^D_0_ transition of bulk Eu^3+^ is severely constrained by the selection rule. This is further supported by the decay dynamics of the ^5^D_0_-^7^F_0_ emission of surface Eu^3+^ in the NCs upon excitation of the Eu^3+^^7^F_0_-^5^D_2_ transition (Figure 4e), which is independent of the increase in Eu^3+^ concentration. Consequently, the entire NC exhibits spectral features mostly originating from surface Eu^3+^ upon these excitations, with these features largely independent from the doping concentration.

The situation is different when bulk Eu^3+^ is effectively excited. Due to the restriction by the selection rule, the de-excitation of the high-lying excited levels of bulk Eu^3+^, which should have been promoted by intensive cross-relaxation, is not significantly accelerated (Figure S14), as demonstrated by the clear recording of luminescence from the ^5^D_1, 2_ levels in the Sr_2_EuF_7_ NCs with an inert-shell grown (Figure 4b). This de-excitation mode, however, is strongly impacted by the presence of surface Eu^3+^. These high-lying levels of bulk Eu^3+^ preferentially de-excite by transferring energy to those of surface Eu^3+^ instead of being sluggishly relaxed. The activation of this de-excitation channel sharply accelerates the luminescence decay of the bulk Eu^3+^^5^D_1_ level (Figure 4f), in striking contrast to the near invariance of its decay behavior in the NCs with an inert-shell grown (Figure S14b). This finding also agrees with the gradual attenuation of the ^5^D_1, 2_ luminescence of bulk Eu^3+^ in the neat core NCs (Figure 4a). The ^5^D_0_ level of surface Eu^3+^ is subsequently populated, thereby generating the corresponding ^5^D_0_ luminescence. This impact brought about by the presence of surface Eu^3+^ becomes more significant with the increase in Eu^3+^ concentration, resulting in the Sr_2_EuF_7_ NCs exhibiting luminescence dominated by the ^5^D_0_-^7^F_2_ emission mainly from surface Eu^3+^ (Figure 4a), regardless of the excitation wavelength chosen (Figures 4c and S15). This finding strikingly challenges the prevailing opinion that Ln^3+^ occupying the lattice site inside the NCs is primarily responsible for the luminescence of Ln^3+^-doped NCs, which calls for attention in future research.

## Conclusions

This work highlights the significance of surface site occupation of Ln^3+^ for the luminescence of Ln^3+^-doped NCs. Sr_2_YF_7_ is identified as a suitable host structure that allows for the distinction between the occupation of Ln^3+^ at bulk and surface sites. The preparation of Sr_2_YF_7_ NCs exclusively featuring surface or bulk Eu^3+^ is achieved through high-temperature surface cation exchange (to obtain surface Eu^3+^-containing NCs) and subsequent epitaxial shell growth experiments (to convert to bulk Eu^3+^-containing NCs), which provide a suitable platform for elucidating their distinct luminescent features. Site-selective spectroscopic analysis reveals a severe coordination environment distortion for Eu^3+^ at the surface of Sr_2_YF_7_, different from that of bulk Eu^3+^ in Sr_2_YF_7_, where Eu^3+^ occupies the Y^3+^ site with D_2_ symmetry. The relaxation of the selection rule caused by a distortion of the local coordination environment confers distinct 4f-4f transition properties on surface Eu^3+^. An in-depth investigation further shows an intensified influence exerted by surface Ln^3+^ in NCs with high doping concentrations, which is demonstrated by steady-state and temporal luminescence analyses, showing that the presence of surface Eu^3+^ sharply accelerates the de-excitation of the high-lying excited levels of bulk Eu^3+^, attributed to the activation of energy transfer between them. Consequently, NCs with high Eu^3+^ concentrations display luminescence largely originating from surface Eu^3+^ in the current case, challenging the prevailing opinion that Ln^3+^ occupying the lattice site inside the NC is primarily responsible for the luminescence of Ln^3+^-doped NCs. These discoveries not only reveal the importance of differently treating the contributions of surface and bulk Ln^3+^ to the luminescence properties of Ln^3+^-doped nanocrystals but also demonstrate the necessity of comprehending the photophysical properties of nanomaterials without any preconceptions, which otherwise may lead to a misunderstanding of the characteristics of targeted materials. An explicit acknowledgment of these aspects aids greatly in developing functional nanomaterials appropriate for nanotechnology research.

## Supporting Information Available

Experimental details, supplemental spectral features of 5%Eu^3+^-doped Sr_2_YF_7_ NCs, detailed emission and excitation spectra of surface and bulk Eu^3+^-containing SrF_2_ and Sr_2_LnF_7_ (Ln^3+^ = Y^3+^, Gd^3+^, Lu^3+^) NCs, spectral features of Eu(III) oleate, and excitation wavelength-dependent emission spectra and luminescence decays of Eu^3+^-doped Sr_2_YF_7_ core and core–shell NCs (PDF)
