# Supplementary material for: Manipulation of Luminescence via Surface Site Occupation in Ln3+-Doped Nanocrystals
Source: J Am Chem Soc. 2024 Apr 16;146(17):11924–31. doi: 10.1021/jacs.4c00052 (PMC11066861; doi:10.1021/jacs.4c00052)
Supplement: Supplementary file 1 — ja4c00052_si_001.pdf [file ja4c00052_si_001.pdf]

Supporting Information for

Manipulation of Luminescence via Surface Site Occupation in Ln^{3+}-Doped Nanocrystals

Rui Shi[1], Litian Lin[2], Zijun Wang[3], Qilin Zou[4], Anja-Verena Mudring[1,5*]

[1] intelligent Advanced Materials, Department of Biological and Chemical Engineering and iNANO, Aarhus University, Aarhus C, 8000 Denmark

[2] State Key Laboratory of Rare Metals Separation and Comprehensive Utilization, Guangdong Provincial Key Laboratory of Rare Earth Development and Application, Institute of Resources Utilization and Rare Earth Development, Guangdong Academy of Sciences, Guangzhou 510651, China

[3] IMRB, Université Paris Est Créteil, INSERM U955, CNRS, EMR 7000, 94010 Créteil, France

[4] Laboratoire de Physique de la Matière Condensée, Ecole Polytechnique, CNRS, IP Paris, 91128, Palaiseau, France

[5] Department of Physics, Umeå University, Linnaeus väg 24, 901 87 Umeå, Sweden.

*Corresponding Author: anja-verena.mudring@bce.au.dk; anja-verena.mudring@umu.se (A.V.M.)

METHODS

Experimental Section: *Chemicals.* $SrCO_3$ (99.9%), trifluoroacetic acid (99%), oleic acid (OA, 90%), 1-octadecene (ODE, 90% tech grade), oleylamine (OM, tech grade), and europium acetate hydrate (99.99%) were purchased from Sigma-Aldrich. Rare-earth oxides (Y_2O_3, Gd_2O_3, Lu_2O_3 and Eu_2O_3, 99.99%) were purchased from HEFA Rare Earth Canada Co. Ltd, Richmond, British Columbia, Canada. Cyclohexane, hexane, and absolute ethanol were purchased from VWR, Denmark. All chemicals were used as received without further purification.

Materials Synthesis. *Preparation of strontium (II) trifluoroacetate (TFA-Sr) and lanthanide (III) trifluoroacetate (TFA-Ln) precursors.* The standard method to prepare the TFA-Ln (or TFA-Sr) precursors is described as follows.[1] Typically, 15 mmol Ln_2O_3 powder, 15 ml 99% trifluoroacetic acid and 15 ml distilled water are added into a 250 ml 2-neck round-bottom flask. This mixture is heated to 90°C until an optically transparent solution is obtained. Generally, different reaction times are required to dissolve all powders (Y_2O_3, 2 h; Lu_2O_3, 6 h, Gd_2O_3 and Eu_2O_3, less than 0.5 h in this case). A condenser is used to avoid the possible evaporation loss of trifluoroacetic acid. After that, the flask is put under dynamic vacuum at a Schlenk line to evaporate water and remaining trifluoroacetic acid, and a white powder of 30 mmol TFA-Ln is then obtained. Next, 45 ml OA and 45 ml ODE are added into the flask, yielding a concentration of TFA-Ln to 0.33 mmol/ml. This mixture is then put under vacuum and slowly heated to 90°C and kept for 30 min. Subsequently, the temperature increases to 120°C and kept for 30 min to disperse all TFA-Ln into the oil phase, and an optically transparent solution is formed. After cooling to room temperature (RT), the TFA-Ln precursor solutions are transferred into vials for later use.

For TFA-Sr, 50 mmol $SrCO_3$ powder, 25 ml 99% trifluoroacetic acid and 5 ml distilled water are added into the flask. The reaction starts immediately after the addition of acid, and all $SrCO_3$ powder completely dissolves within 5 min. The flask is then put under vacuum to evaporate water and remaining trifluoroacetic acid, and a white powder of 50 mmol TFA-Sr is obtained. Next, 75 ml OA and 75 ml ODE are added into the flask, yielding a concentration of TFA-Sr to 0.33 mmol/ml. This mixture is then put under vacuum and slowly heated to 120°C and kept for 1 h, and an optically transparent solution is formed. After cooling back to RT, the TFA-Sr precursor is transferred into vials for later use.

Preparation of Eu(III) oleate used for surface exchange. 5 mmol europium acetate hydrate and 15 ml OA and 15 ml ODE are added into the flask. The flask is then put under vacuum and slowly heated to 150°C kept for 1 h to dissolve all europium acetate, and an optically transparent solution is formed with the concentration of Eu(III) oleate to 0.17 mmol/ml. After cooling back to RT, the Eu(III) oleate dispersed in 1-octadecene is transferred into vials for later use.

Synthesis of Eu^{3+}-doped Sr_2YF_7 nanocrystals (NCs). The series of Eu^{3+}-doped Sr_2YF_7 NCs are prepared by thermal decomposition method.[1] In a typical procedure, 4 mmol TFA-Sr (12 ml) and 2 mmol TFA-Ln (Ln = Y and Eu) (6 ml) precursor solutions are added into a 250 ml 3-neck round-bottom flask according to nominal chemical formulae of $Sr_2Y_{1-x}Eu_xF_7$ (x = 0.01 to 1). Additional 3 ml OA, 17 ml ODE and 4 ml OM are added to the flask to reach a total amount of 12 ml OA, 26 ml ODE, and 4 ml OM. Next, the mixture is put under vacuum at a Schlenk line and heated to 90°C at roughly 400 rpm stirring. The mixture is kept at 90°C for 10 min, and then Ar and vacuum are cycled for three times to remove any residual oxygen and moisture to prevent the incorporation of such impurities that may lead to radiationless decay of the excited state. Subsequently, the solution is heated to 300°C under gentle Ar flow for roughly 15 min. The reaction is kept at 300°C for 30 min. Then the heating mantel is removed, and the mixture is cooled to RT naturally. The viscous solution obtained is transferred to two 50 ml centrifuge tubes, and roughly 30 ml mixed solvent (hexene: ethanol (v/v) = 1:2) is added into each tube to precipitate the product

NCs. After centrifugation at 8000 rpm for 10 min, a sediment is obtained at the bottom of tube. The washing process involves dispersing these products in 15 ml cyclohexene, adding 30 ml absolute ethanol to precipitate, and centrifuging at 8000 rpm. After the washing step, nominally 2 mmol of Eu^{3+}-doped Sr_2YF_7 NCs are obtained. These product NCs are dispersed and stored in 20 ml cyclohexane for later use.

Synthesis of Sr_2GdF_7, Sr_2LuF_7 and SrF_2 NCs. The preparation of Sr_2GdF_7, Sr_2LuF_7 and SrF_2 NCs follows the same experimental procedures as the synthesis of Sr_2YF_7 NCs.

Inert-shell growth procedure for Eu^{3+}-doped Sr_2YF_7 NCs (Eu^{3+}-doped Sr_2YF_7@Sr_2YF_7). The shell-growth procedure follows a layer-by-layer hot-injection protocol. Firstly, different amounts of TFA-Sr and TFA-Y precursor solutions are mixed with each other (v/v = 2:1) under continuous stirring at RT to reach the concentration of Sr_2YF_7 shell precursor to 0.11 mmol/ml for later use. In a typical procedure to introduce the core, 1 ml cyclohexane solution nominally containing 0.1 mmol Eu^{3+}-doped Sr_2YF_7 core NCs is added into a 100 ml 3-neck round-bottom flask with 5 ml OA and 5 ml ODE. This mixture is put under vacuum and heated to 90°C at roughly 400 rpm stirring for 5 min. After that, Ar and vacuum are cycled for three times to remove cyclohexane and oxygen, and the solution is heated to 300°C under gentle Ar flow for 15 min. To create the shell, a certain amount of well-mixed shell precursor is transferred into an injection syringe and injected through a septum into the flask when temperature of reaction system reaches to 270°C. The injection amount is set to be 1 ml for each injection, and the interval time between each injection is 3 min. Depending on the different amounts of used shell precursor, different reaction times are required. After the completion of the precursor injection, the solution is continued to react for 5 min further and then cooling back to RT naturally. The same sample washing procedure as for the neat core (see above) is adopted, and the resultant NCs are dispersed and stored in 10 ml cyclohexane.

Experimental details for the Y^{3+}-to-Eu^{3+} surface exchange and successive epitaxial shell growth. First, 1 mmol undoped Sr_2YF_7 NCs are prepared. For that, 2 mmol TFA-Sr (6 ml) and 1 mmol TFA-Y (3 ml) precursor solutions are added into a 100 ml 3-neck round-bottom flask. 1.5 ml OA, 8.5 ml ODE and 2 ml OM are added to the flask to reach a total amount of 6 ml OA, 13 ml ODE, and 2 ml OM. Next, the mixture is heated to 90°C at roughly 400 rpm stirring and put under vacuum at a Schlenk line. The mixture is kept at 90°C for 10 min, and then Ar and vacuum are cycled for three times to remove any residual oxygen and moisture. The solution is then heated to 300°C under gentle Ar flow for roughly 15 min and kept at 300°C for 30 min. After this stage, an aliquot (roughly 2 ml) is extracted from the reaction mixture using a glass syringe and stored in a glass vial for further treatment. Next, 0.2 mmol Eu(III) oleate dispersed in 1-octadecene (1.2 ml) is hot-injected through a septum into the flask in one shot, which triggers the Eu^{3+} exchange at the surface of Sr_2YF_7 NCs. (0.02 mmol Eu(III) oleate dispersed in 1-octadecene (0.12 ml) is hot-injected into the flask in the low concentration Eu^{3+} surface exchange experiment, other experimental procedures remain unchanged.) The moment of injection is defined as the time reference of 0 min. Aliquots (roughly 2 ml each time) are then extracted from the reaction mixture with the exchange durations of 1, 5, 9, 15, and 20 min. After this stage, the pre-prepared inert Sr_2YF_7 shell precursor with the concentration of 0.11 mmol/ml is hot-injected into the reaction system shot-by-shot, with the injection amount of 1 ml per injection, and a 3 min interval time between each injection. Aliquots are then extracted from the reaction mixture when the nominal molar ratio of luminescent-core and inert-shell compositions reach to 1:1, 1:3, and 1:7, respectively, and then the solution is cooled to RT naturally. These aliquots are washed as described above, and the resultant NCs are dispersed and stored in 5 ml cyclohexane.

Experimental details for Eu^{3+} surface exchange and successive epitaxial shell growth in the systems of Sr_2GdF_7, Sr_2LuF_7 and SrF_2. The same experimental procedures are followed as those for the Y^{3+}-to-Eu^{3+} exchange and epitaxial shell growth, except that TFA-Gd, TFA-Lu and TFA-Sr precursor solutions are used during the synthesis of undoped NCs as well as the inert-shell growth.

Characterization. The phase purity of studied samples was examined on a STOE Stadivari X-ray diffractometer with Cu Kα radiation (λ = 0.15405 nm). Infrared spectra of samples were measured with a Bruker Alpha-P ATR-spectrometer (Bruker AXS, Karlsruhe, Germany). The program OPUS (Bruker, Ettlingen, Germany) was used for data evaluation. Transmission electron microscopy images of NCs were taken on with an FEI Titan Themis microscope operating at 300 kV with a Falcon3 EC 4k/4k Direct Detection Electron camera. Emission and excitation spectra of samples dispersed in cyclohexane were measured at RT on a spectrofluorometer (FluoroMax-P, Horiba) with a 150 W ozone-free xenon arc-lamp and an R928P photomultiplier tube as the detector. An Edinburgh FLS1000 combined fluorescence steady-state and lifetime spectrometer was used for collecting the high-resolution excitation and emission spectra and the luminescence decays of Eu^{3+}-doped NCs in the solid form at RT, equipped with a 450W Xe900 xenon lamp and a 60 W μF2 flash lamp with a pulse width of 1−2 μs as the excitation sources, respectively, and a thermoelectric cooled (−22 °C) R928P photomultiplier (Hamamatsu) for detection. The collected luminescence signals of the samples have undergone corrections, as has the background signal subtraction. The mono-exponential function is used to estimate the lifetime of the luminescence from the $^5D_0$ level of bulk Eu^{3+}, whose luminescence decay closely obeys the exponential characteristic. For surface Eu^{3+} whose decay feature of the luminescence from $^5D_0$ level deviates from the exponential characteristic, the average lifetime is evaluated using the formula: $\tau_{average} = \int_0^\infty \frac{I(t)}{I(0)} dt$, where I(0) and I(t) represent the initial intensity and that at time *t*, respectively.[2]

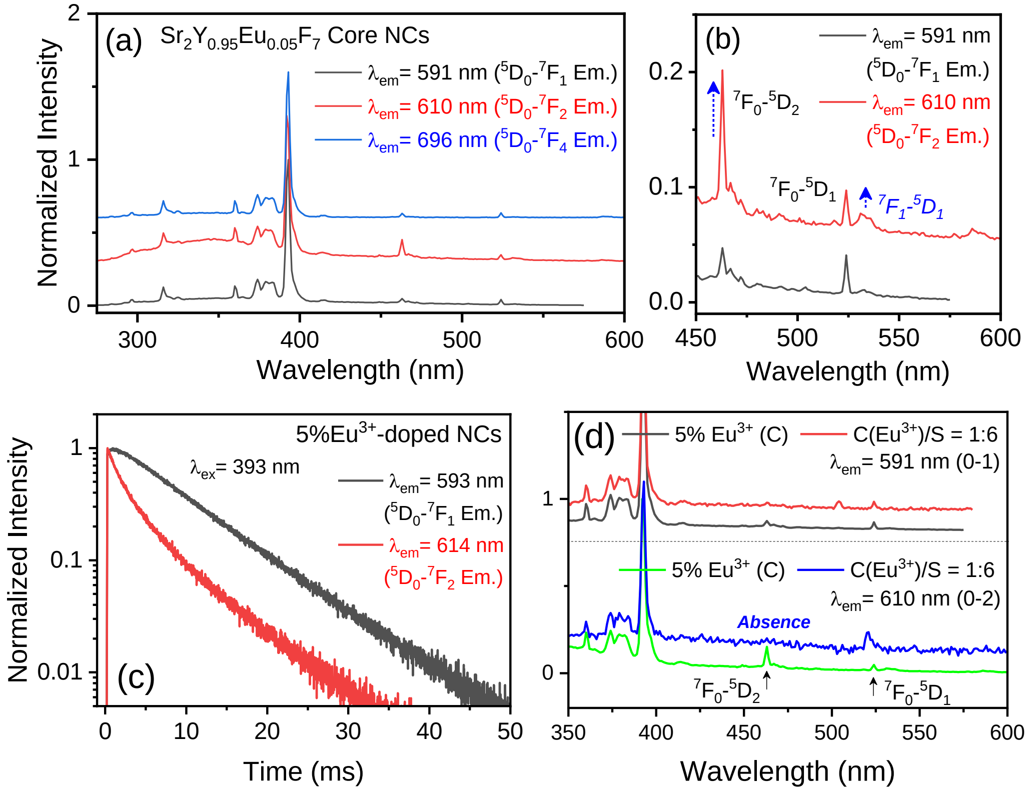

Figure S1. (a) Normalized excitation spectra of $Sr_2Y_{0.95}Eu_{0.05}F_7$ recorded at different emission wavelengths and (b) enlargement in the wavelength range of 450 to 600 nm. (c) Luminescence decay curves of $Sr_2Y_{0.95}Eu_{0.05}F_7$ sample by monitoring 593 nm (corresponding to the Eu^{3+} $^5D_0$-$^7F_1$ transition) and 614 nm (corresponding to the Eu^{3+} $^5D_0$-$^7F_2$ transition) emissions upon 393 nm excitation (corresponding to the Eu^{3+} $^7F_0$-$^5L_6$ transition). (d) Comparison of the normalized excitation spectra of 5%Eu^{3+}-doped core and core-shell Sr_2YF_7 NCs (with a nominal molar ratio of luminescent-core and inert-shell compositions equal to 1:6) monitoring different emission wavelengths.

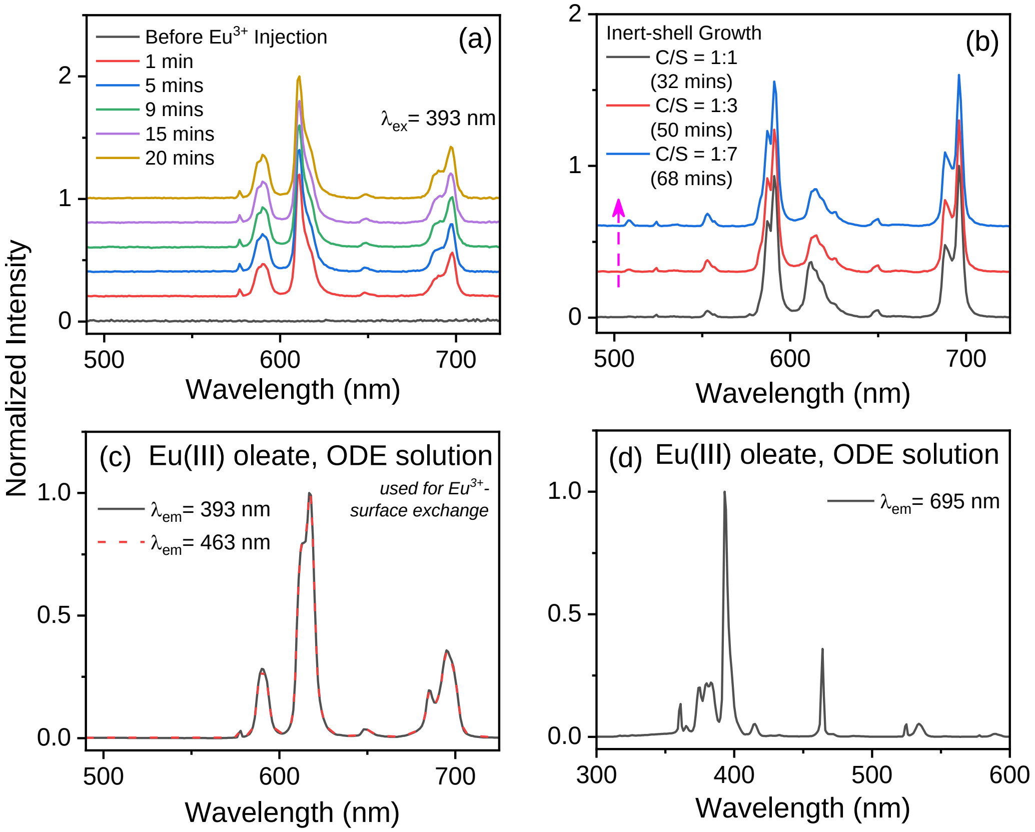

Figure S2. (a) Normalized emission spectra of aliquots of Eu^{3+}/Y^{3+} surface exchanged Sr_2YF_7 NCs successively extracted from the reaction mixture with different exchange durations and (b) those with inert-shell growth upon 393 nm excitation. (c) Normalized emission spectra of Eu(III) oleate dispersed in 1-octadecene used as the Eu^{3+} exchange source during the experiment upon different excitations. (d) The corresponding excitation spectrum of Eu^{3+}-oleate recorded at 695 nm emission (corresponding to the Eu^{3+} $^5D_0$-$^7F_4$ transition).

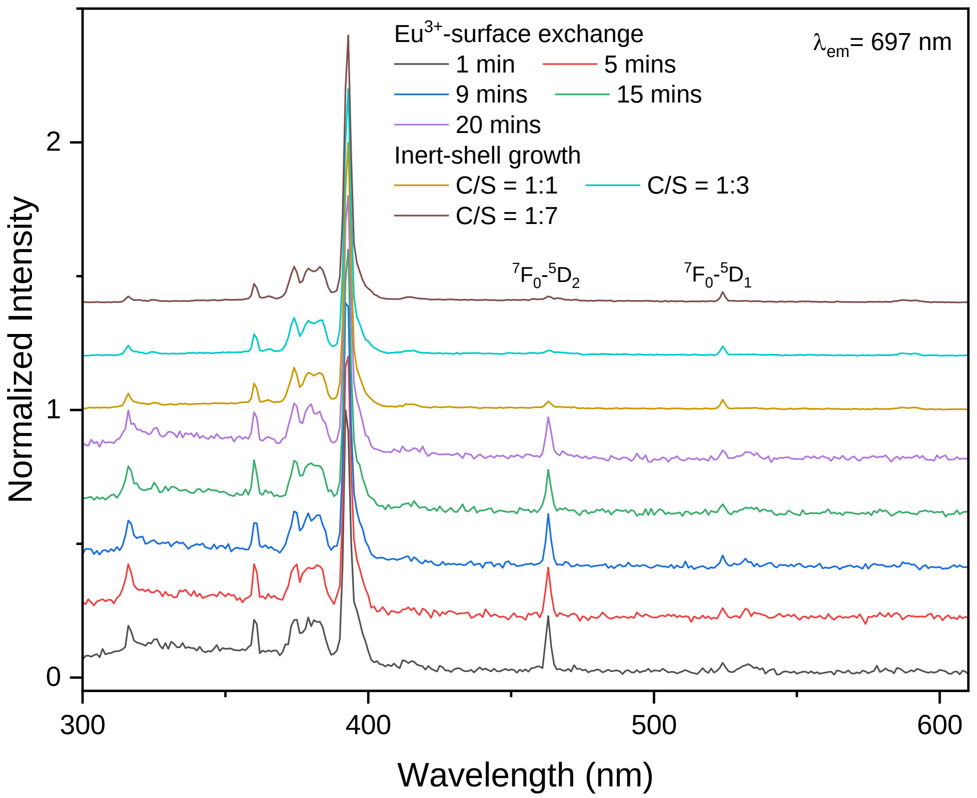

Figure S3. Normalized excitation spectra of aliquots successively extracted from the reaction mixture by monitoring with 697 nm emission (corresponding to the Eu^{3+} $^5D_0$-$^7F_4$ transition).

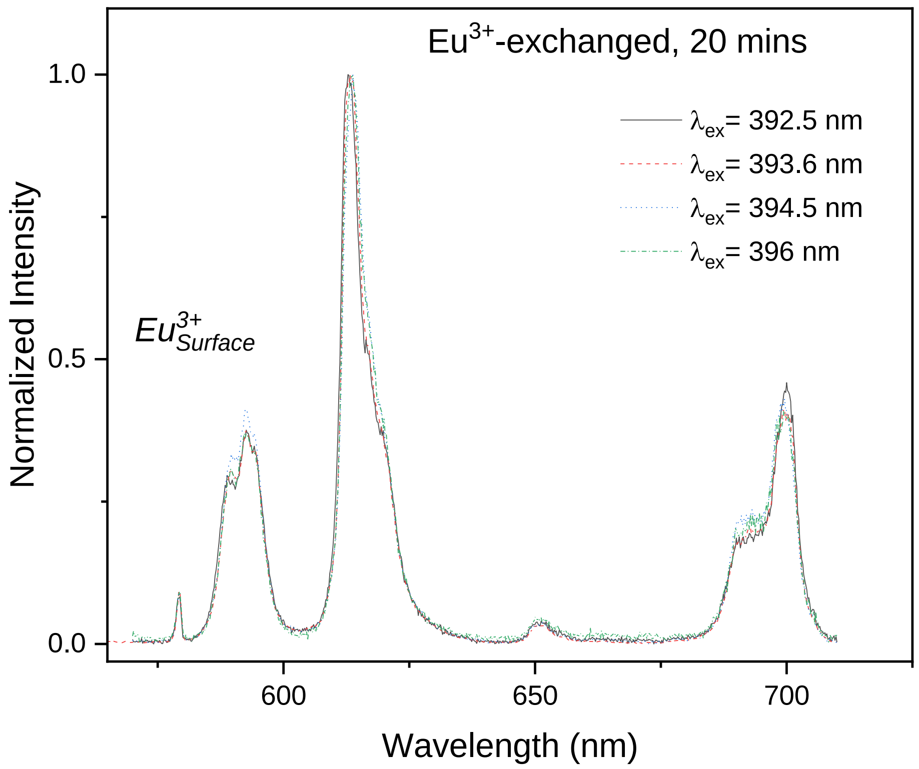

Figure S4. Normalized excitation spectra of surface Eu^{3+} in Eu^{3+}/Y^{3+} surface exchanged Sr_2YF_7 upon different excitations.

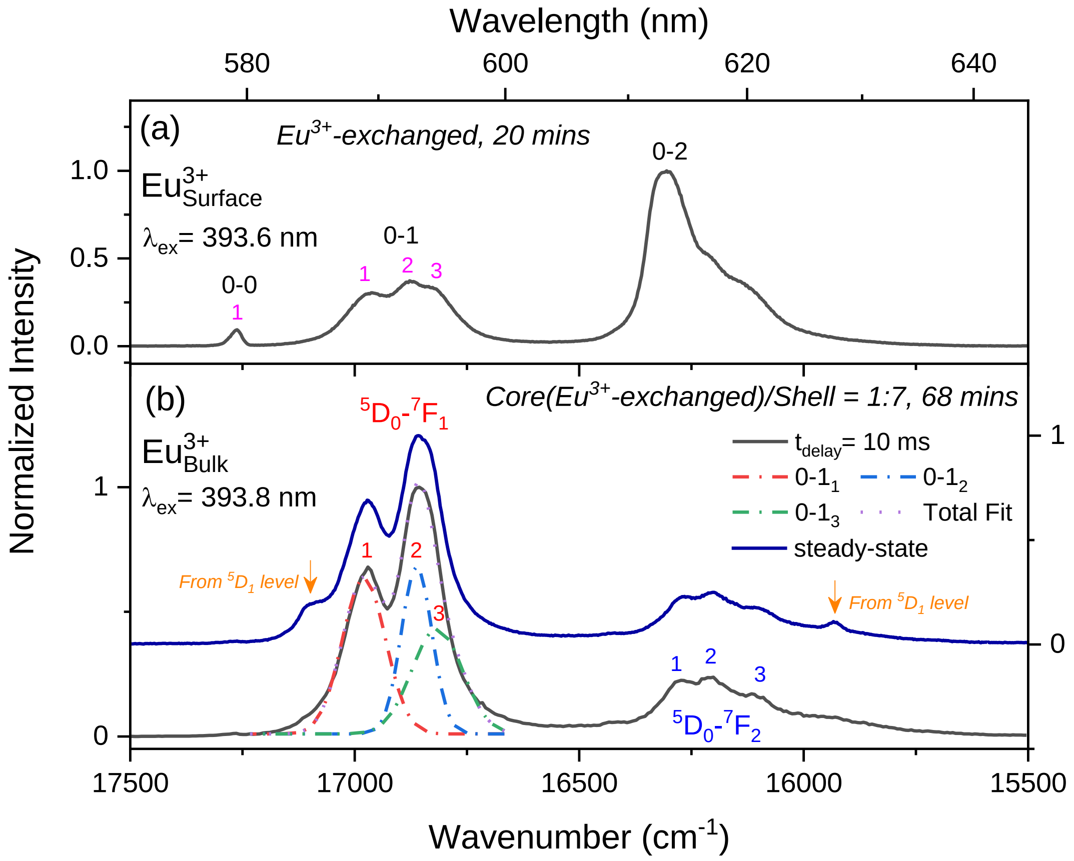

Figure S5. (a) High-resolution emission spectrum of surface Eu^{3+} in Eu^{3+}/Y^{3+} surface exchanged Sr_2YF_7 upon 393.6 nm excitation (corresponding to the Eu^{3+} $^7F_0$-$^5L_6$ transition). A threefold splitting of the $^5D_0$-$^7F_1$ transition is observed, with one $^5D_0$-$^7F_0$ line recorded at around 579 nm. (b) High-resolution emission spectra of bulk Eu^{3+} in core-shell Eu^{3+}/Y^{3+} surface exchanged Sr_2YF_7 upon 393.8 nm excitation (corresponding to the Eu^{3+} $^7F_0$-$^5L_6$ transition). Both threefold splitting of the $^5D_0$-$^7F_1$ and $^5D_0$-$^7F_2$ transitions are observed in the spectra. No signal of the $^5D_0$-$^7F_0$ line can be detected in the spectrum when delaying the signal acquisition (t_{delay} = 10 ms).

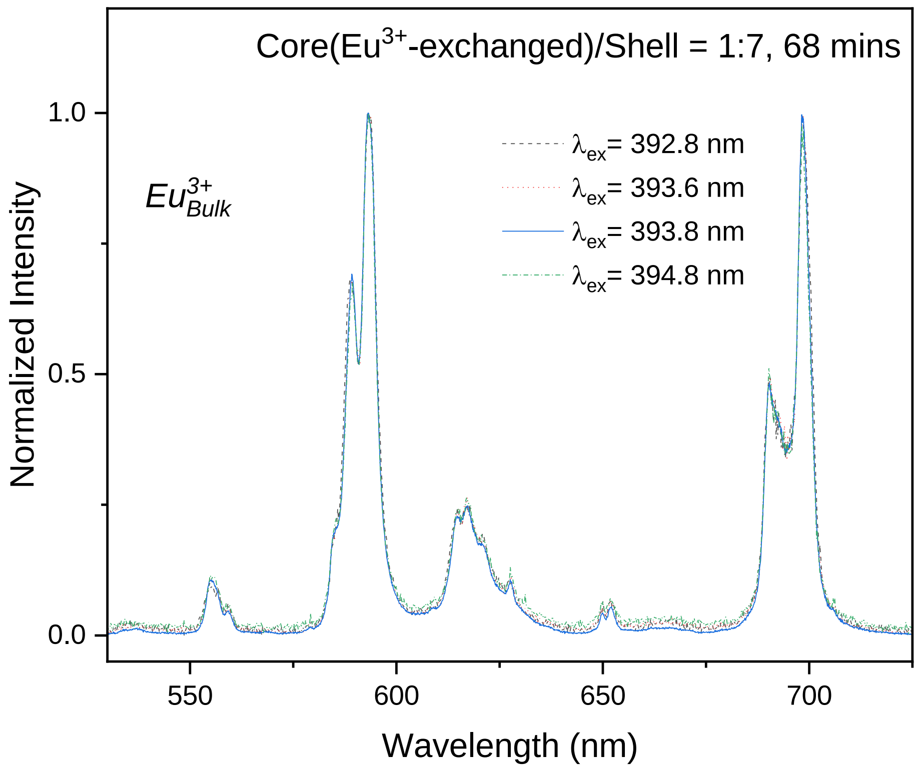

Figure S6. Normalized excitation spectra of bulk Eu^{3+} in core-shell Eu^{3+}/Y^{3+} surface exchanged Sr_2YF_7 upon different excitations. Comparison of the emission spectra of bulk Eu^{3+}-containing NCs recorded by fine-tuning the excitation wavelength allows to exclude the presence of different bulk Eu^{3+} sites with significantly different local environments, revealing the sole cationic site occupation of bulk Eu^{3+} in the nano-crystal.

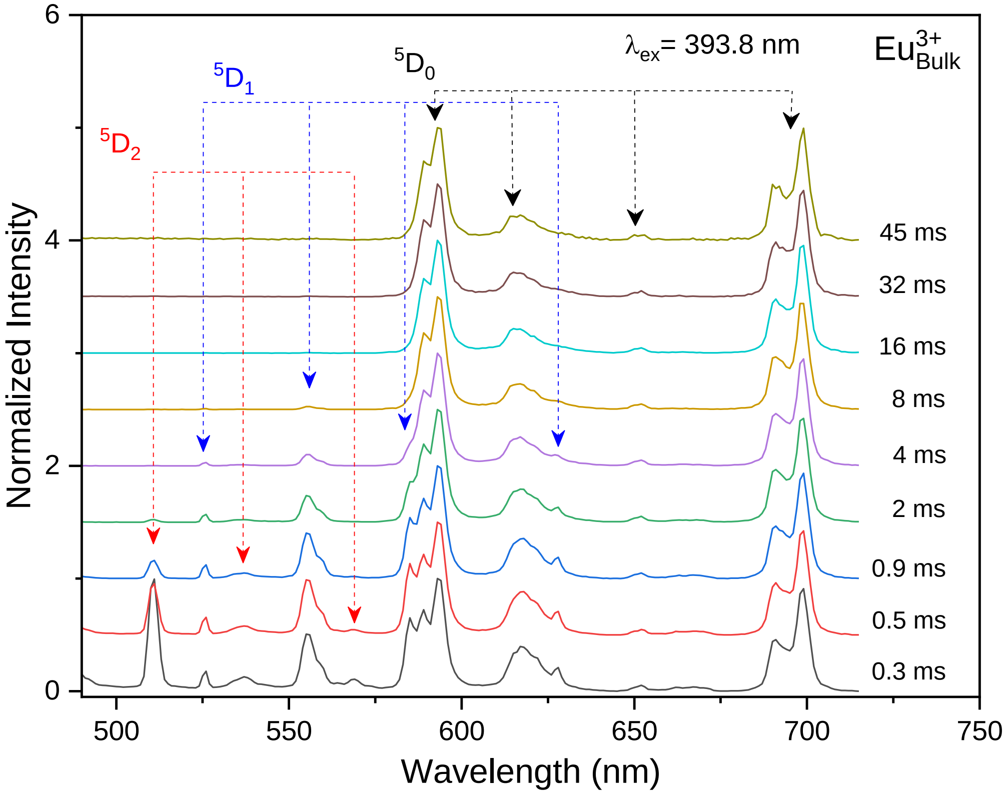

Figure S7. Normalized time-resolved emission spectra of bulk Eu^{3+} in core-shell Eu^{3+}/Y^{3+} surface exchanged Sr_2YF_7 with delay times ranging from 0.3 ms to 45 ms upon 393.8 nm excitation. The luminescence signals from different excited levels of bulk Eu^{3+} are marked by arrows in different colors.

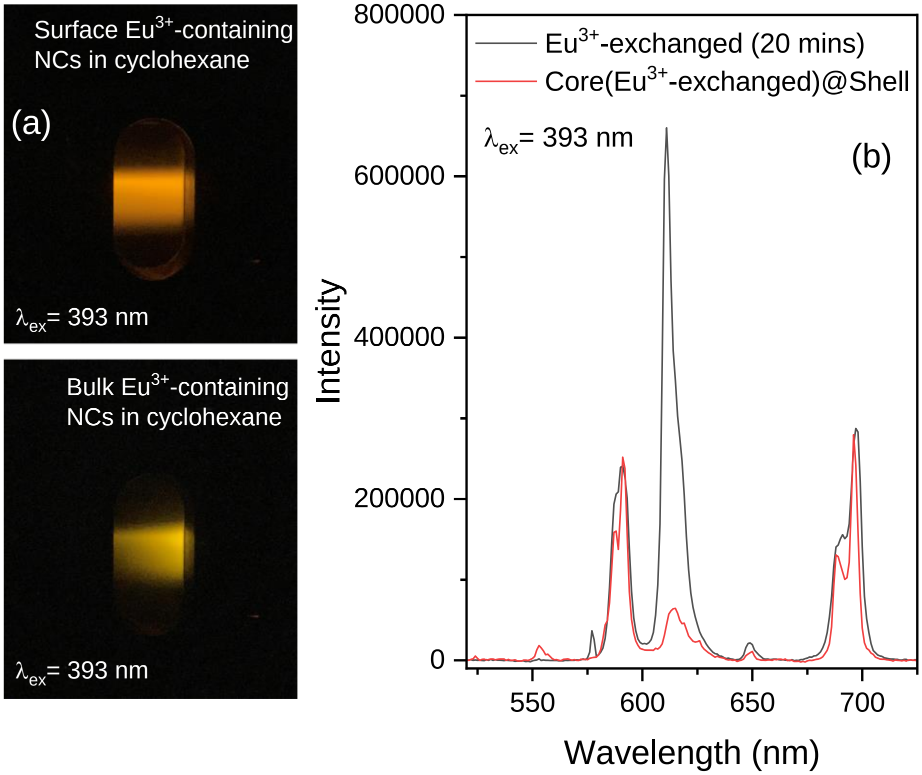

Figure S8. (a) Comparison of the luminescence photographs of surface Eu^{3+}-containing, and bulk Eu^{3+}-containing Sr_2YF_7 NCs dispersed in cyclohexane as collected upon 393 nm excitation. These photographs were taken with an identical camera setup. The particle concentration in these two specimens is set to be nominally the same (0.1 mmol/ml), with the doping concentration of Eu^{3+} in the NCs largely constant. (b) The corresponding emission spectra of samples.

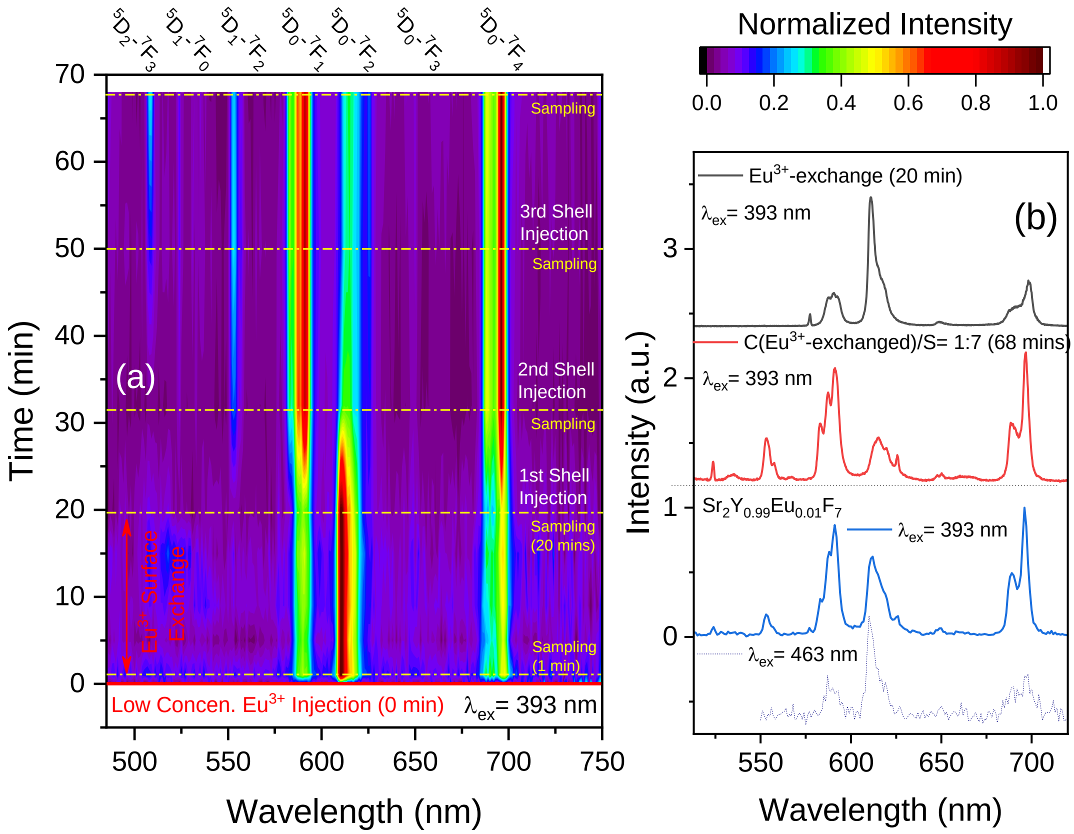

Figure S9. (a) The color contour of the normalized emission spectra of Eu^{3+} in NCs extracted from the reaction mixture at different stages in the low-concentration Eu^{3+} surface exchange experiment. The moment of injection of Eu^{3+} source is defined as the time reference of 0 min. Compared to the case of the Y^{3+}-to-Eu^{3+} surface exchange experiment as shown in Figure 2, the amount of Eu(III) oleate which was one order of magnitude lower than used before (from 0.2 mmol to 0.02 mmol) dispersed in 1-octadecene (0.12 ml) was injected into the flask to trigger the Eu^{3+} surface exchange. Other experimental procedures remained unchanged. (b) Comparison of the emission spectra of Eu^{3+} residing at the surface or in the interior of NCs as collected from the low-concentration Eu^{3+} surface exchange experiment as well as the emission spectra of $Sr_2Y_{0.99}Eu_{0.01}F_7$ NCs upon different excitations.

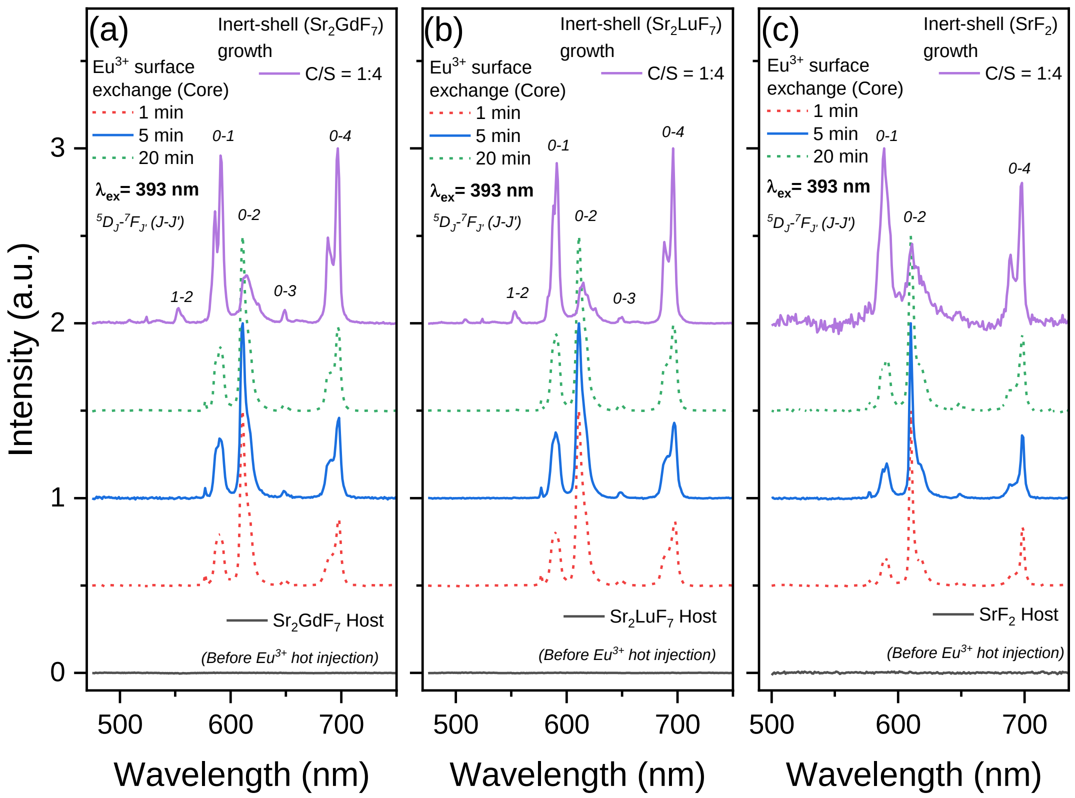

Figure S10. Comparison of the emission spectra of undoped, surface Eu^{3+}-containing and bulk Eu^{3+}-containing (a) Sr_2GdF_7, (b) Sr_2LuF_7 and (c) SrF_2 NCs as collected from the Eu^{3+} surface exchange experiments upon 393 nm excitation.

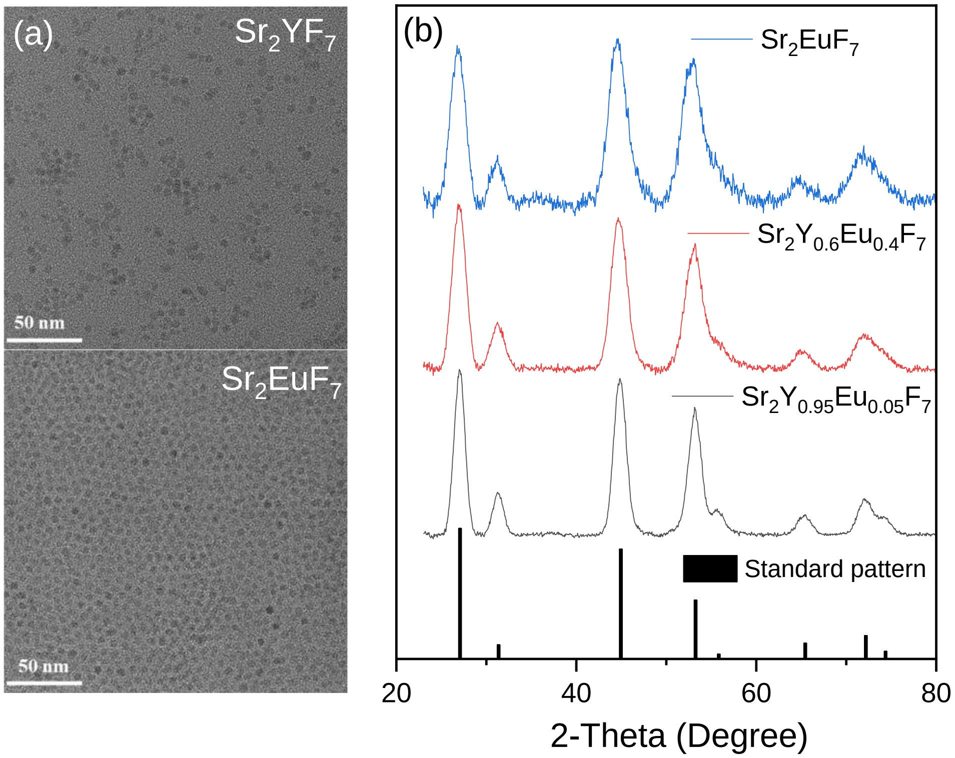

Figure S11. (a) TEM images of surfactant-capped Sr_2YF_7 and Sr_2EuF_7 NCs. (b) PXRD patterns of different Eu^{3+}-doped NCs.

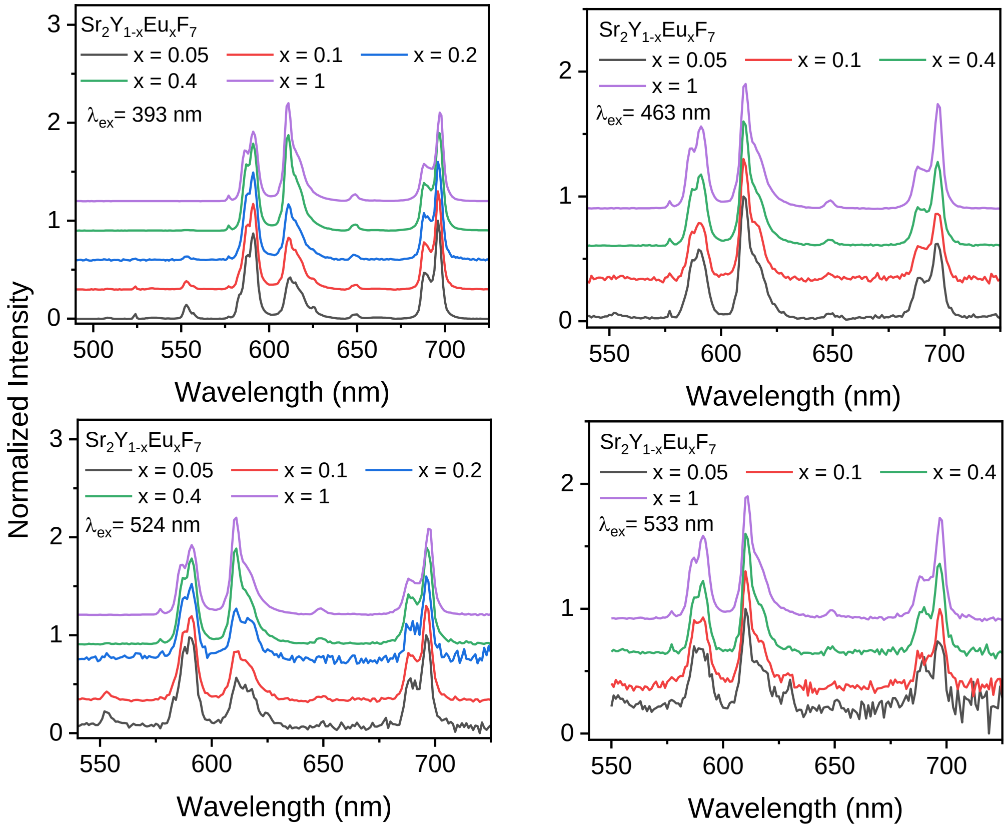

Figure S12. Normalized concentration-dependent emission spectra of Eu^{3+}-doped Sr_2YF_7 NCs upon different excitations.

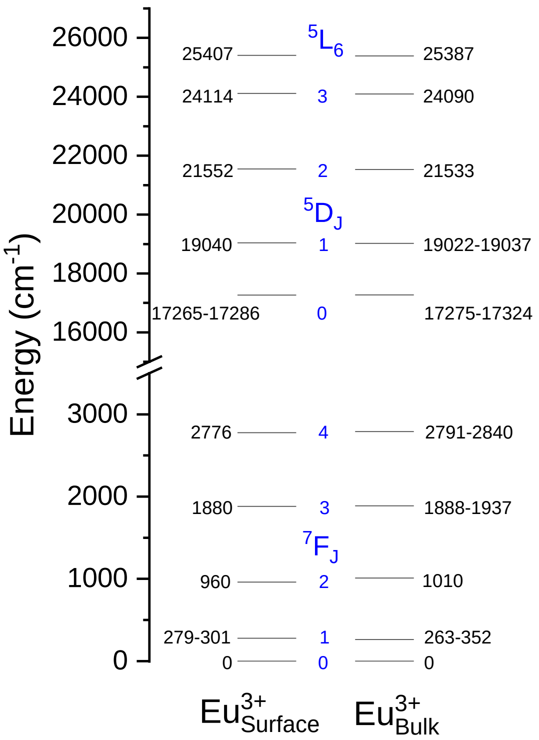

Figure S13. Schematic energy level diagrams for surface and bulk Eu^{3+} in Eu^{3+}-doped Sr_2YF_7. The energies of different levels of Eu^{3+} obtained from the high-resolution excitation and emission spectra are clearly marked. The slight variation in value is due to the determination employing different 4f-4f transition energies in the system.

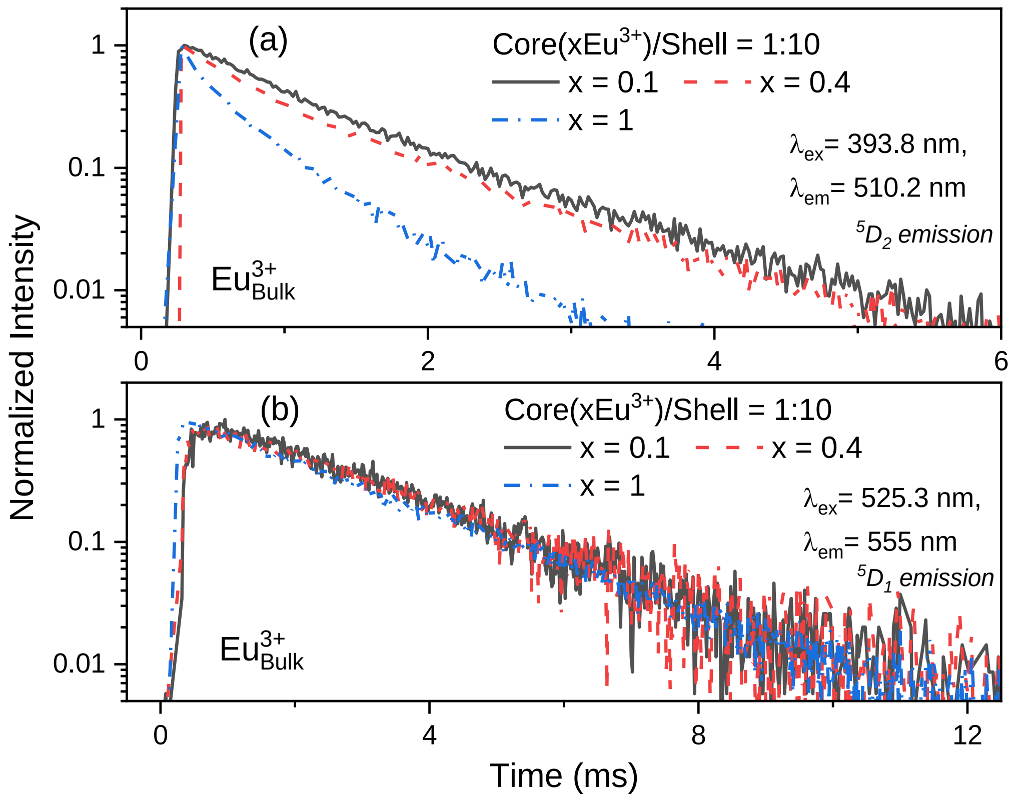

Figure S14. (a) Luminescence decay dynamics of the $^5D_2$ and (b) the $^5D_1$ excited levels of bulk Eu^{3+} in the core-shell Eu^{3+}-doped Sr_2YF_7 NCs (with a nominal molar ratio of core and shell compositions equal to 1:10) with different Eu^{3+} concentrations.

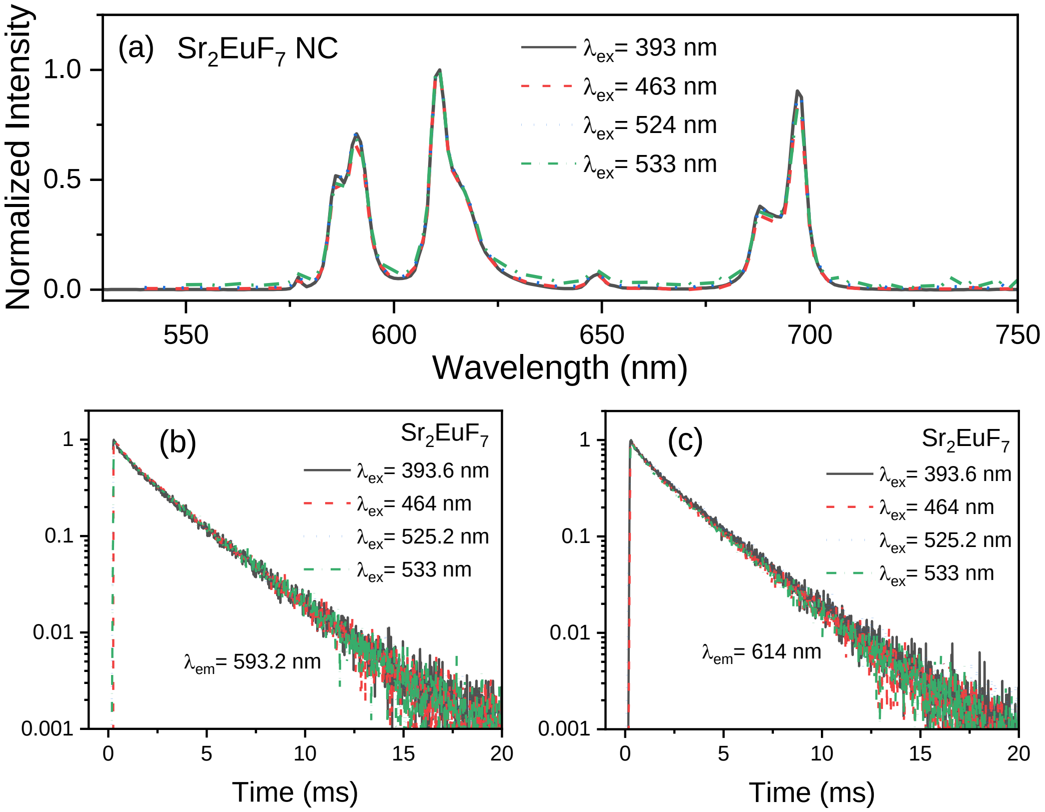

Figure S15. (a) Normalized emission spectra of Sr_2EuF_7 NCs upon different excitations. (b) Decay curves of the Eu^{3+} $^5D_0$-$^7F_1$ and (c) the $^5D_0$-$^7F_2$ emissions of Sr_2EuF_7 upon different excitations.

REFERENCES

1. Fischer, S.; Mehlenbacher, R. D.; Lay, A.; Siefe, C.; Alivisatos, A. P.; Dionne, J. A., Small Alkaline-Earth-based Core/Shell Nanoparticles for Efficient Upconversion. *Nano Lett.* **2019,** *19* (6), 3878-3885.
2. Zatryb, G.; Klak, M. M., On the choice of proper average lifetime formula for an ensemble of emitters showing non-single exponential photoluminescence decay. *Journal of Physics: Condensed Matter* **2020,** *32* (41), 415902.
